# Transcranial alternating current stimulation: a review of the underlying mechanisms and modulation of cognitive processes

**DOI:** 10.3389/fnhum.2013.00279

**Published:** 2013-06-14

**Authors:** Christoph S. Herrmann, Stefan Rach, Toralf Neuling, Daniel Strüber

**Affiliations:** ^1^Experimental Psychology Lab, Center of excellence Hearing4all, Department for Psychology, Faculty for Medicine and Health Sciences, Carl von Ossietzky Universität, Ammerländer HeerstrOldenburg, Germany; ^2^Research Center Neurosensory Science, Carl von Ossietzky UniversitätOldenburg, Germany

**Keywords:** alpha, EEG, electroencephalogram, gamma, oscillations, transcranial direct current stimulation, transcranial alternating current stimulation, transcranial magnetic stimulation

## Abstract

Brain oscillations of different frequencies have been associated with a variety of cognitive functions. Convincing evidence supporting those associations has been provided by studies using intracranial stimulation, pharmacological interventions and lesion studies. The emergence of novel non-invasive brain stimulation techniques like repetitive transcranial magnetic stimulation (rTMS) and transcranial alternating current stimulation (tACS) now allows to modulate brain oscillations directly. Particularly, tACS offers the unique opportunity to causally link brain oscillations of a specific frequency range to cognitive processes, because it uses sinusoidal currents that are bound to one frequency only. Using tACS allows to modulate brain oscillations and in turn to influence cognitive processes, thereby demonstrating the causal link between the two. Here, we review findings about the physiological mechanism of tACS and studies that have used tACS to modulate basic motor and sensory processes as well as higher cognitive processes like memory, ambiguous perception, and decision making.

## Introduction

**Figure 1:**
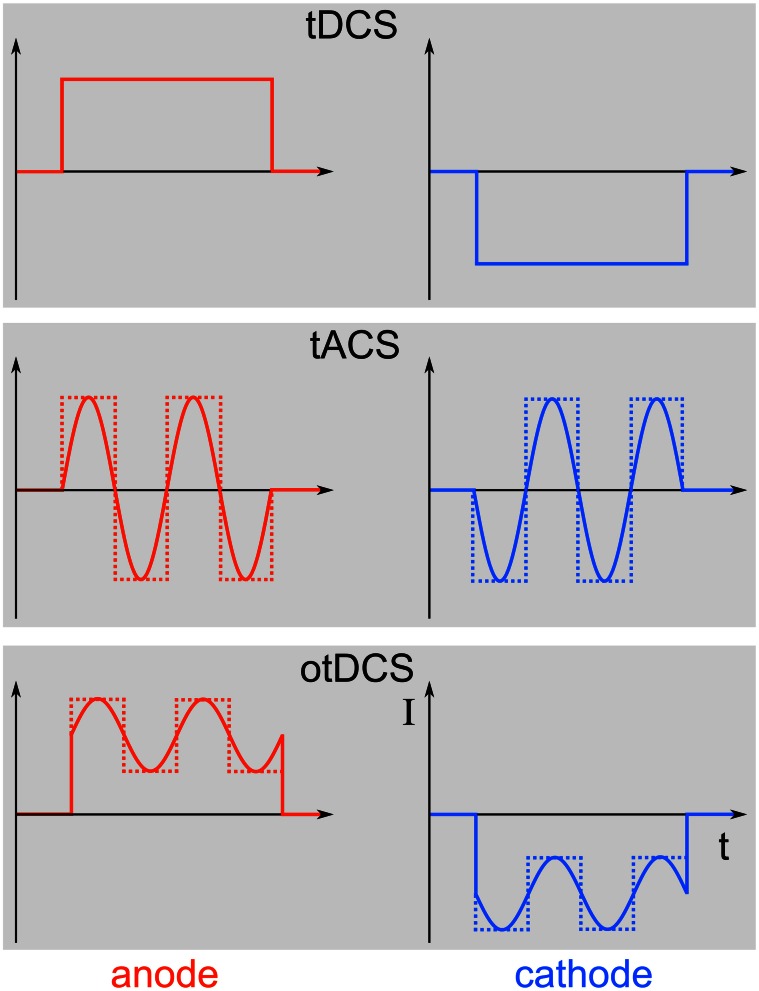
**Different stimulation paradigms. Top:** During tDCS, a direct current will be switched on for a duration of typically a few minutes. **Middle:** In contrast, tACS uses alternating currents for stimulation that can either be sinusoidal (solid line) or rectangular (dotted line). **Bottom:** Combining tDCS and tACS results in oscillatory tDCS (otDCS), where the alternating current is superimposed onto a direct current. The alternating current can again be sinusoidal or rectangular.

Transcranial electric stimulation (tES) is the superordinate term for a class of non-invasive brain stimulation techniques comprising direct current (DC), alternating current (AC), and random noise (RN) stimulation (Paulus, 2011). The principle of using electric currents to stimulate the human body and brain is not new (see Priori, 2003 for a review). The applied currents can either be constant over time, as is the case with transcranial direct current stimulation (tDCS), or they can alternate at a certain frequency, which is referred to as transcranial alternating current stimulation (tACS). Stimulation with a RN frequency spectrum is known as transcranial random noise stimulation (tRNS). Here, we want to focus on tACS, since this method is particularly well suited to modulate physiologically relevant brain oscillations in a frequency-specific manner. Oscillations and DCs can be combined to more complex waveforms. If DC and AC are combined for transcranial stimulation, this is referred to as oscillatory tDCS (otDCS, Groppa et al., 2010). The AC does not need to be sinusoidal but may as well be rectangular or have even more complex shapes (Figure 1).

**Figure 2:**
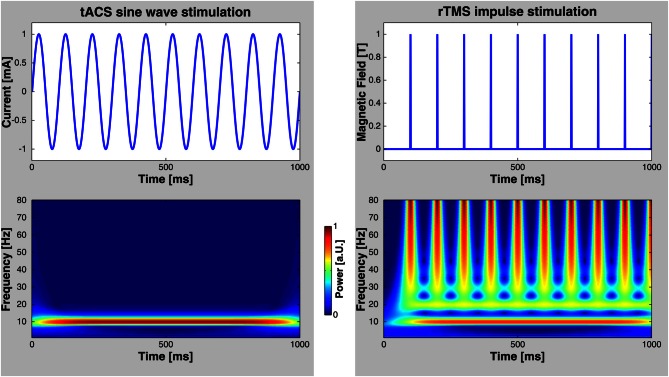
**Comparison of rTMS and tACS. Left:** tACS uses sinusoidal currents which are restricted to one frequency as shown by a time-frequency wavelet transform. **Right:** rTMS, however, spans a wide range of frequencies in addition to the frequency of repetition. Note, that these diagrams depict only the stimulation currents/fields—not possible artifacts that may be elicited in the human brain.

Numerous elegant studies during the last few decades have demonstrated a close association between brain oscillations and cognitive functions (for reviews, see Başar et al., 2001; Engel et al., 2001; Herrmann et al., 2004). The link has, however, always been established by correlating oscillatory brain activity with specific cognitive processes. Therefore, the issue of whether brain oscillations reflect a fundamental mechanism in cortical information processing or just an epiphenomenon is still unresolved. It has been argued that if oscillations were essential for any cognitive function, then this function should be altered by selectively interfering with these oscillations (Sejnowski and Paulsen, 2006). This has been considered to be a very difficult question to answer empirically in healthy humans until recently (Rees et al., 2002). One possibility to address this important issue is to study abnormal oscillatory activity in patients with neuropsychiatric disorders (Herrmann and Demiralp, 2005; Schnitzler and Gross, 2005; Uhlhaas and Singer, 2006). For example, it has been shown that the degree of cognitive deficits in patients with attention deficit hyperactivity disorder (ADHD) is correlated with an amplitude reduction of gamma-band oscillations in a memory paradigm (Lenz et al., 2008). However, complex diseases are usually not the result of one single symptom like disturbed gamma oscillations. Therefore, such studies provide evidence for an association between clinical symptoms and deviances in brain oscillations but do not provide causal links. Probing a causal role of oscillations for cognition has been promoted by recent developments of non-invasive human brain stimulation techniques that allow for driving brain oscillations within the range of observable, physiologically relevant frequencies. For one such technique—repetitive transcranial magnetic stimulation (rTMS)—the capability to entrain brain oscillations has been demonstrated recently (Thut et al., 2011). Compared to otDCS and tACS, rTMS is spatially more precise and neurons are excited directly by each TMS pulse (Thut et al., 2011). On the one hand, rTMS delivers brief bursts of about 100 μs duration that can be repeated at the frequency that is believed to be responsible for a certain cognitive effect. On the other hand, as depicted in Figure 2, repetitive bursts span a wide range of frequencies. Thus, care must be taken when ascribing rTMS-induced effects to the repetition frequency of rTMS. A recent article nicely mentions the criteria required to consider an effect to be based upon rhythmic entrainment of brain oscillations (Thut et al., 2011). In the case of tACS it is less likely to entrain brain oscillations other than the stimulation frequency, since the sinusoidal currents are strictly bound to only one frequency. Nevertheless, the finding of frequency specific rTMS effects on behavior (e.g., Romei et al., 2011) demonstrates that rTMS mainly entrains oscillations at the stimulation frequency and not at the broad-band responses of the single pulses.

In addition, tACS does not generate sounds that could interfere with the experimental paradigm and can be applied in the absence of somatosensory sensations—thus allowing for easy control conditions.

## Physiological mechanism of tACS

**Figure 3:**
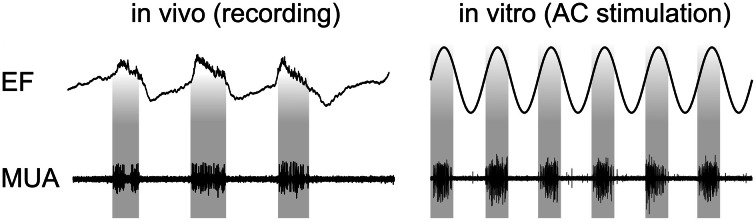
**Physiological mechanisms of tACS. Left:** *In vivo* recordings in ferrets show that spontaneous neuronal activity seen in MUA synchronizes to certain phases of LFPs. **Right:** Stimulating slices of cortex electrically with sinusoidal currents results in a similar synchronization. Interestingly, the inter-burst frequency of the spontaneously occurring activity can be speeded up and slowed down resulting in neural entrainment [adapted from Fröhlich and McCormick (2010)].

Recently, the physiological mechanisms that underlie the observed tACS effects have been revealed via intracranial recordings in animals (Fröhlich and McCormick, 2010). The authors stimulated ferrets intracranially and simultaneously recorded local field potentials (LFPs) and multiunit activity (MUA). Before stimulating the animals, *in vivo* recordings demonstrated that neuronal spikes in MUAs were synchronized to the oscillatory LFPs (Figure 3, left). Subsequently, cortical slices were stimulated *in vitro* and MUA was recorded simultaneously revealing that weak sinusoidal voltages (≤0.5 V/m) were able to elicit spiking activity (Figure 3, right). Intriguingly, the spiking activity synchronized to different driving frequencies, suggesting that neuronal firing can be entrained to the electrically applied field (not shown here). Furthermore, Fröhlich and McCormick (2010) were able to demonstrate that steep transient voltage changes lead to stronger neural firing than slow transients albeit reaching the same voltage maximum [see supplemental information of Fröhlich and McCormick (2010)]. This indicates that not only absolute voltage levels determine neural firing but the temporal dynamics of voltage changes are important.

From that study, however, it was not clear whether weak currents can also penetrate the skull and still have similar effects upon neural activity. Ozen et al. (2010) have addressed this question by stimulating rats with electrodes on the surface of the skull while recording neural activity intracranially. These authors were able to show that an intracranial electric field as low as ~1 V/m was sufficient to synchronize neural firing to a specific phase of the extracranially applied sinusoidal current. The current that has to be applied extracranially to achieve this electric field depends upon multiple parameters, such as skull thickness, electrode placement, and the like. This issue will be addressed in Section Modeling current flow.

A recent experiment in humans revealed that changes of cortical excitation depend non-linearly upon the intensity of tACS (Moliadze et al., 2012). Primary motor cortex was stimulated with tACS at 140 Hz while motor evoked potentials (MEPs) were simultaneously recorded in response to single TMS pulses. Low stimulation intensities of 0.2 mA resulted in cortical inhibition as indexed by increased motor thresholds. High intensities of 1 mA resulted in decreased thresholds, i.e., excitation. Intermediate intensities of 0.6 and 0.8 mA had no effect on motor threshold. This seems to indicate that inhibitory neurons are more susceptible to electric stimulation and are stimulated already at lower intensities. Excitatory neurons are less susceptible and require stronger stimulation but dominate the inhibitory neurons leading to a net effect of excitation. At intermediate intensities, inhibitory and excitatory effects cancel each other out.

An important step for tACS was to show its effectiveness in modulating oscillatory brain activity in humans. In this context, Zaehle et al. (2010) demonstrated that tACS applied at participants' individual EEG alpha frequency resulted in an enhancement of the EEG alpha amplitude after 10 min of stimulation. EEG was recorded offline, i.e., three minutes before and after applying tACS. After tACS, spectral power was significantly increased specifically in the range of the individual alpha frequency (IAF ~10 ± 2 Hz) as compared to before tACS, indicating that this stimulation method can interfere with ongoing brain oscillations in a frequency-specific way despite its low amplitude and its transcranial application. A recent study by Neuling et al. (2013) replicated and extended the findings of Zaehle et al. (2010) by showing that the tACS-induced alpha amplitude enhancement remains present for at least 30 min after stimulation offset. Interestingly, alpha amplitude was only enhanced when the effective intracranial alpha tACS amplitude exceeded the endogenous alpha amplitude (eyes-open condition), but not when the former was weaker than the latter (eyes-closed condition).

Further insights into the effect of tACS can be expected from simultaneously recording hemodynamic responses with functional magnetic resonance imaging (fMRI), as has been done for brief impulses of TES (Brocke et al., 2008). While this procedure seems challenging for tDCS due to hemodynamic artifacts, it appears feasible for tACS (Antal et al., 2013).

### Modeling current flow

**Figure 4:**
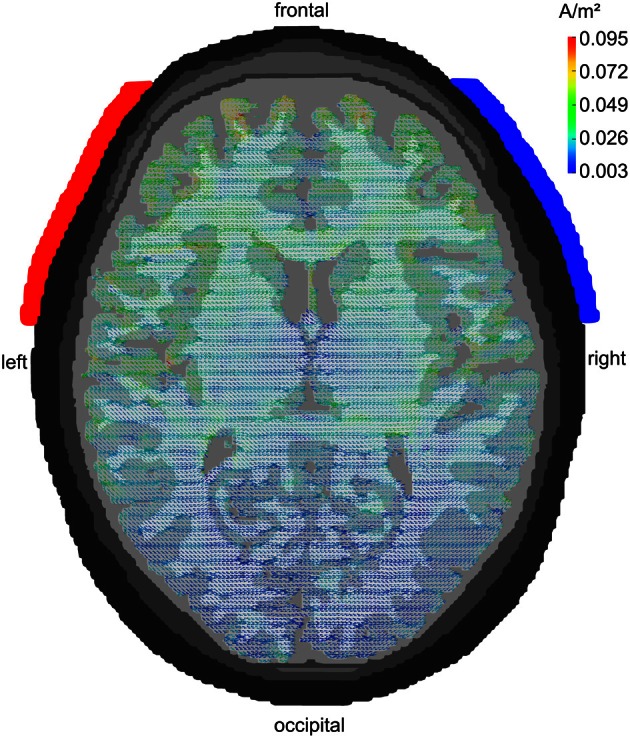
**Modeling tACS-induced intracranial current flow**. Axial view of a human brain visualizing the current density distribution of tDCS/tACS applied at electrode locations F7 (anode, red) and F8 (cathode, blue). A clear maximum in anterior brain areas can be seen. Current densities are 20 times stronger in frontal as compared to occipital cortex. However, tDCS/tACS with large electrodes is not as focal as TMS. Reprinted from Neuling et al. (2012b) with permission of the authors.

A series of modeling studies has investigated how much of the weak extracranially applied current (typically around 1 mA in tACS) arrives intracranially. Early studies have used spherical head models to address this issue (Miranda et al., 2006). Later approaches used more realistically shaped head models that were derived from MRI recordings (Holdefer et al., 2006; Wagner et al., 2007). A large amount of the current is short-circuited by the well-conducting skin. Nevertheless, significant current densities can be modeled intracranially that result from the extracranial stimulation. Miranda et al. (2006) demonstrated that 2 mA of tDCS results in 0.1 A/m^2^ of intracranial current density^1^ corresponding to an electric field of 0.22 V/m. Neuling et al. (2012b) used a very fine-grained finite element model to show that 1 mA of tDCS/tACS applied to human visual cortex results in an intracranial current density of 0.1 A/m^2^ amounting to a cortical electric field of 0.417 V/m when assuming a gray matter conductivity of 0.24 S/m (Figure 4). Compared to the thresholds for synchronizing neural spikes to electric fields derived from intracranial recordings in animals (0.5–1 V/m) this would suggest that 1 mA of tDCS/tACS would be near or below threshold whereas 2 mA would be well above threshold.

Recent modeling studies demonstrate that focality of tDCS/tACS can be significantly enhanced when multiple small electrodes, e.g., EEG electrodes, are used instead of the typical 5 × 7 cm sponge electrodes (Faria et al., 2009; Dmochowski et al., 2011). However, even the usage of small electrodes suffers from the fact that at least two electrodes are required to apply a current to the human head. Therefore, two foci of current density result from the use of equally sized anode and cathode or from a small stimulation electrode and a larger return electrode. This problem can be overcome by using a so-called 4 × 1 ring electrode configuration (Datta et al., 2009). This montage uses four electrodes arranged in a ring for one polarity of stimulation, e.g., cathode, and another single electrode placed in the middle of the ring for the other polarity, e.g., anode. A single region of current density results from this electrode arrangement. The stimulated region can be located in a specific brain area by appropriate electrode placement.

Electric stimulation of brain tissue in animals revealed that the axon–especially the axon hillock–but not the soma is susceptible to electric fields (Nowak and Bullier, 1998). In addition, it has been demonstrated that electric fields along an axon are much more effective than those perpendicular to the axon (Ranck, 1975). This has led to the idea of differentiating between the currents that flow radial with respect to the cortical surface and those that flow tangential (Miranda et al., 2012). Since pyramidal cells are oriented perpendicularly to the surface of the cortex and large parts of their axons in white matter are oriented tangentially to the cortex surface, it is tempting to directly assign radial electric fields to the soma of pyramidal cells and tangential ones to the axon. However, due to the anisotropy of white matter fibers, such a simplification may be premature.

### Computational network models

Beside their above mentioned physiological work, Fröhlich and McCormick (2010) also set out to simulate neural responses to direct and sinusoidal currents. They used the Hodgkin-Huxley model (Hodgkin and Huxley, 1952) in order to simulate how a network of 400 pyramidal neurons and 64 inhibitory interneurons responds to applied DC and AC fields. Importantly, they were able to demonstrate that the frequency of the applied field determined the degree of entrainment of the neural oscillations. If the driving frequency was close to the intrinsic frequency, membrane voltages showed strong periodic fluctuations. In contrast, if the external field differed significantly from the intrinsic frequency, no such entrainment was observed. These findings are in line with theoretical considerations of entrainment (Pikovsky et al., 2003).

Using the tACS parameters from Zaehle et al. (2010) as a reference, Merlet et al. (2013) simulated scalp EEG activity under tACS compared to no stimulation. Effects of tACS on EEG mean alpha power were modeled for different frequencies from 4 to 16 Hz in steps of 1 Hz. The strongest increase in alpha power was found at 10 Hz tACS, with progressively decreasing effects for the neighboring frequencies (8/9 Hz and 11/12 Hz). Outside the 8–12 Hz band, no significant tACS effects were found. These simulation findings correspond to the experimental results in humans by Zaehle et al. (2010). Furthermore, the modeled results demonstrated that alpha tACS is most efficient at the intrinsic frequency (10 Hz for the model).

**Figure 5:**
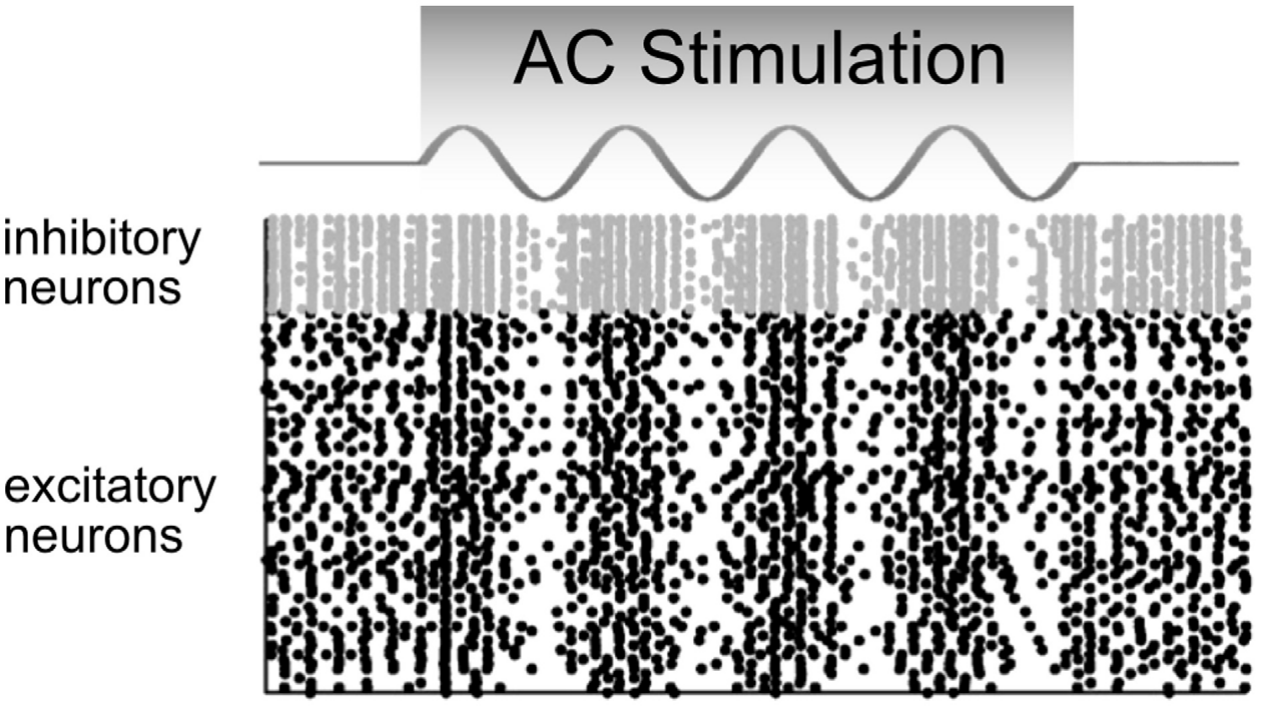
**Model predictions of how a network of neurons would behave in response to AC stimulation**. The firing rates of inhibitory (gray) and excitatory (black) neurons are up- and down-regulated in phase with the AC current. In these raster plots, each dot represents a neural spike. Adapted from Reato et al. (2010).

Reato et al. (2010) used a simplified version of the Hodgkin Huxley model in which 800 excitatory and 200 inhibitory hippocampal neurons were modeled according to the integrate-and-fire model of Izhikevich (2003). The results demonstrated that:

1. DC stimulation mainly affects the firing rate.
2. AC stimulation up- and down-regulates the firing rate in an oscillatory manner without changing the average firing rate over a longer time interval (Figure 5).
3. AC stimulation at the frequency of endogenous oscillations mainly affects spike timing.
4. Even low amplitudes of electrical stimulation corresponding to a cortical electric field of 0.2 V/m result in enhanced coherence between spikes and the driving oscillation.

Interestingly, these simulations demonstrate a neural mechanism which could be responsible for the cross-frequency coupling that has been found in electrophysiological recordings (Jensen and Colgin, 2007). It has been repeatedly demonstrated that the phase of theta oscillations modulates the amplitude of gamma oscillations (Canolty et al., 2006; Demiralp et al., 2007), i.e., theta oscillations spread out cortically and their phase modulates gamma amplitudes. If the cortex were stimulated electrically at a frequency in the theta range, these artificial theta oscillations could spread out in the same way as physiological fields have been shown to do. The phase of these oscillations could then modulate the amplitude of gamma oscillations.

**Figure 6:**
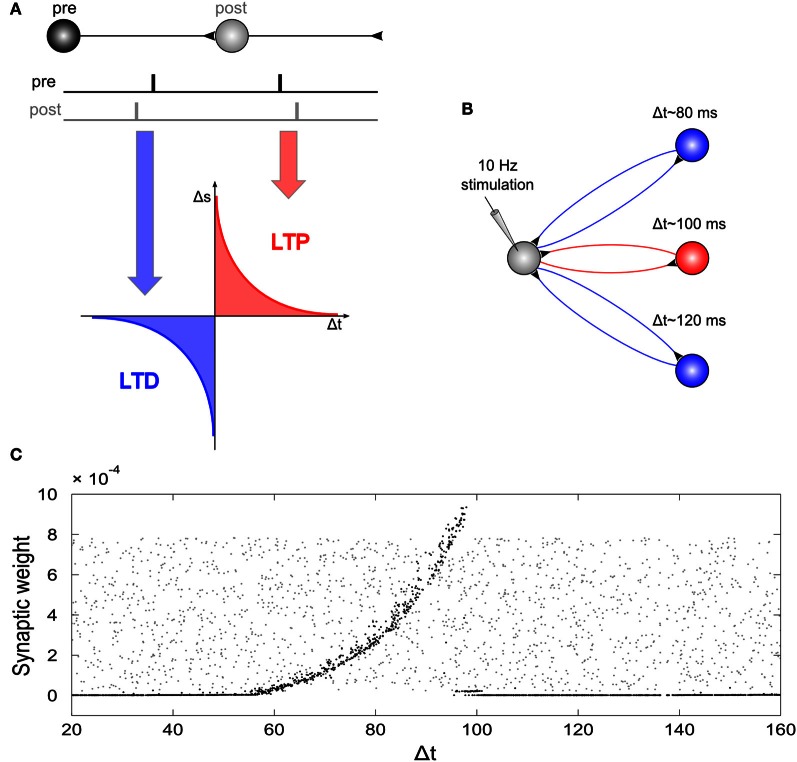
**Network simulation of tACS. (A)** Spike timing dependent plasticity: synaptic weights are increased if a post-synaptic potential follows a pre-synaptic spike (long-term potentiation, LTP) and decreased if a post-synaptic potential occurs prior to a pre-synaptic spike (long-term depression, LTD). **(B)** Schematic illustration of the network: A driving neuron establishes a recurrent loop with each neuron of a hidden layer. The total synaptic delay, Δt, (i.e., the sum of both delays of the loop) varied between 20 and 160 ms. The driving neuron was stimulated with a spike train of 10 Hz repetition rate. **(C)** Synaptic weights of the back-projection as a function of the total synaptic delay of the recurrent loops: Gray dots display synaptic weights at the start of the simulation, black dots represent synaptic weights after the end of simulation. External stimulation of the driving neuron at 10 Hz resulted in increased weights for recurrent loops with a total delay between 60 and 100 ms, and dramatically reduced synaptic weights for loops with total delays outside this interval. Note, that the highest synaptic weights are observed at 100 ms, i.e., for loops with a resonance frequency near the stimulation frequency. Reprinted from Zaehle et al. (2010) with permission of the authors.

Due to the strong artifact that tACS produces during the time of stimulation, so far, effects on electrophysiology have only been shown for EEG after stimulation as compared to before stimulation (Marshall et al., 2006; Zaehle et al., 2010). However, the above stimulation experiments in animals and the simulation experiments in “silicon cells” explain only how electric stimulation affects electrophysiology at the time of stimulation. In order to simulate also the after-effects of their EEG experiment, Zaehle et al. (2010) used a neural network composed of Izhikevich neurons. They used a single neuron that was driven by an external current and 2500 neurons that were connected to the driven neuron by axons with variable delay times resulting in 2500 resonance loops with different resonance frequencies (Figure 6). During stimulation with a 10 Hz spike train, spike-timing-dependent-plasticity (STDP) modulated those synapses that were incorporated into loops with resonance frequencies close to the frequency of the driving force (100 ms~10 Hz). This finding suggests that synaptic plasticity was responsible for the observed after-effect of tACS. Along the same lines, neuroplastic changes have also been proposed as the mechanism underlying tACS after-effects by other authors (Antal and Paulus, 2012).

## Motor and cognitive functions modulated by tACS

### Motor processes

Probing tACS/otDCS effects on the primary motor cortex bears the advantage to objectively measure changes of cortico-spinal excitability with MEPs after TMS. Compared to, e.g., phosphene ratings in the visual domain, MEPs do not rely on subjective experience of the participants. MEPs are used as a dependent variable that is usually compared from a baseline before stimulation to one or more time points during and after stimulation. Encouraging evidence with regard to motor excitability and behavior has been reported by existing studies, whereas conclusive electrophysiological results are largely missing, due to a general lack of concurrent EEG measurements. Exceptions are studies by Antal et al. (2008) and Pogosyan et al. (2009), which combined tACS and EEG. Therefore, effects on behavior, excitability, and electrophysiological effects will be reviewed in separate sections.

#### Effects on motor cortex excitability

The first study utilizing tACS and anodal/cathodal otDCS to investigate effects on the motor cortex was conducted by Antal et al. (2008). This exploratory study intended to compare oscillatory TES protocols to established constant current TES protocols. The authors analyzed MEP amplitudes before and after tACS/otDCS with different durations and frequencies. No effects on MEPs were found. Subsequent studies indicated that the weak after effects could be attributed to the stimulation parameters, e.g., comparatively short stimulation durations (2–10 min) and weak stimulation intensities (tACS: 0.25 A/m^2^; otDCS: 0.16 A/m^2^; current density in the electrode^2^). For example, otDCS studies with at least 10 min of stimulation and mean intensities of 0.63 A/m^2^ were able to reveal after-effects (Bergmann et al., 2009; Groppa et al., 2010). Depending on the polarity, cortico-spinal excitability could be increased or decreased (Groppa et al., 2010). However, these effects do not differ from control conditions utilizing tDCS, suggesting that the DC portion of the stimulation currents caused the observed effects. Additionally, with a maximal intensity of 0.62 A/m^2^, polarity dependent effects have only been demonstrated for tDCS but not for otDCS, indicating that not the maximal intensity but the overall current (mean intensity: tDCS, 0.62 A/m^2^; otDCS: 0.31 A/m^2^) was relevant for the effects (Groppa et al., 2010). Unfortunately, EEG was not recorded in these studies to differentiate effects of otDCS compared to tDCS.

Studies using tACS revealed bidirectional excitability shifts both during and after stimulation. Short tACS with different frequencies (5, 10, 20, 40 Hz; 90 s; 0.14 A/m^2^) revealed that only during 20 Hz tACS motor excitability increased (Feurra et al., 2011a). Likewise, a study by Schutter and Hortensius (2011) yielded no increased excitability after 10 Hz tACS (10 min; 0.298 A/m^2^) but after a combined frequency stimulation (5 Hz followed by 20 Hz; 5 min each; 0.298 A/m^2^), although the specific contributions of the applied frequencies cannot be differentiated. Vice versa, tACS with 15 Hz (20 min; 0.80 A/m^2^) decreased excitability after stimulation (Zaghi et al., 2010). Moliadze et al. (2010) applied tACS at frequencies outside traditional EEG frequency bands in the so called ripple range (80, 140, and 250 Hz; 10 min; 0.63 A/m^2^). Only stimulation with 140 Hz resulted in sustained excitability enhancement of the motor cortex for up to 1 h after stimulation. Even higher frequencies (1000, 2000, and 5000 Hz; 10 min; 0.20 A/m^2^) are also able to modulate cortical excitability (Chaieb et al., 2011). Increased excitability has been reported during and up to 90 min after stimulation. This effect was most pronounced for 5000 Hz and was interpreted as an interference with neuronal membrane excitation but not entrainment of neural oscillations.

#### Behavioral effects

Diverse behavioral effects after applying different tACS frequencies raise the possibility to causally link specific frequencies to distinct functions. A significant role takes the beta rhythm (15–30 Hz) as the “natural frequency” of motor regions (Rosanova et al., 2009). Beta synchrony correlates with slower voluntary movement (Gilbertson et al., 2005). In the same way, tACS with 20 Hz slowed down voluntary movement, indicating a causal relationship (Pogosyan et al., 2009; Joundi et al., 2012; Wach et al., 2013).

Applying two different tACS frequencies allows to dissociate frequency-behavior relationships. Joundi et al. (2012) found that 20 Hz tACS (5 s; ~0.26 A/m^2^) slowed down voluntary movement, but 70 Hz tACS with the same parameters increased the performance, extending correlative studies which found increased gamma band activity (30–70 Hz) during voluntary movement (Muthukumaraswamy, 2010). Besides the 20 Hz slowing effect, Wach et al. (2013) observed an increased behavioral variability after 10 Hz tACS with the same parameters (10 min, 0.29 A/m^2^). The authors attributed the 10 Hz effect to a disruption of an internal pacemaker represented by activity in the alpha range. Interestingly, both effects occurred at different time points: the 20 Hz effect was found immediately after stimulation, but not after 30 min, conversely to the 10 Hz effect.

#### Electrophysiological effects

A major drawback of the present studies on the effects of tACS/otDCS is the lack of electrophysiological evidence. This is rather unfortunate in light of the assumption that tACS and otDCS interact with oscillatory brain activity. Although studies on the effects of tACS/otDCS on motor processes imply the promising advantage to demonstrate changes in the EEG, so far, only few studies reported electrophysiological results. Stimulation with different frequencies (1, 10, 15, 30, 45 Hz) yielded no EEG effects after tACS/otDCS with different frequencies (Antal et al., 2008). But, as mentioned above, weak stimulation intensity could explain absent effects.

Future studies, combining tACS/otDCS and EEG, could be helpful in two different aspects. First, changes in EEG frequency bands, e.g., parameters like power and synchrony, could be related to the previously reported behavioral effects, further strengthening the assumption of a causal oscillation-behavior relationship. Second, by comparing the specific effects after tACS/otDCS and tDCS, contributions of the constant and time varying part of the stimulation could be disentangled. Particular attention should be paid to frequencies that are predominant in the EEG during specific tasks, because tACS/otDCS might only be effective to entrain physiologically relevant rhythms (Thut et al., 2011).

### Sensory processing

#### Phosphenes induced by tACS: cortical or retinal origin?

The earliest effect of tACS on the human visual system was reported by Kanai et al. (2008). These authors studied the influence of different tACS frequencies on the detection of phosphenes induced by tACS over visual cortex (stimulation electrode of 3 × 4 cm placed 4 cm above the inion; reference electrode of 9 × 6 cm placed at the vertex). Participants were stimulated at 5 different intensities (125, 250, 500, 750, and 1000 μA) with 12 frequencies ranging from 4 to 40 Hz in randomized order with each frequency being applied 5 s in the light and 5 s in the dark, consecutively. After stimulation, participants had to rate the phosphenes in both conditions with respect to a standard phosphene induced by tACS with 16 Hz at 1000 μA in the light (maximum current density under the stimulation electrode 0.83 A/m^2^). The results indicated that the effectiveness of tACS indeed varied with stimulation frequency and that this effect was moderated by the surrounding light conditions. In a dark room, stimulation was most effective in the range of 10–12 Hz, whereas in a light room, phosphene thresholds were lowest for stimulation in a frequency range between 14 and 20 Hz. In a second experiment, these results were replicated by measuring phosphene detection thresholds. The authors explained their results with the change of dominant oscillation frequencies in the natural EEG with respect to different light conditions: in darkness, the most prominent oscillations are found in the alpha range (8–12 Hz), which are, however, suppressed and replaced by higher frequencies in the light.

Although Kanai et al. (2008) assumed that their finding of tACS-induced phosphenes results from an excitatory tACS effect on parts of the visual cortex, this view has been questioned subsequently by Schwiedrzik (2009). This author referred to earlier work demonstrating that AC can reliably excite retinal ganglion cells and that the frequency at which this effect occurs depends upon the dark adaptation of the retina (Schwarz, 1947). Phosphenes are absent when retinal ganglion cells are inhibited due to pressure on the eyeball (Rohracher, 1935). In line with this argumentation favoring a retinal phosphene origin, it has been demonstrated that a more anterior placement of tACS electrodes over fronto-central areas leads to stronger phosphenes than a more posterior placement over occipito-central regions (Schutter and Hortensius, 2010). These findings have been replicated recently by Kar and Krekelberg (2012). However, Paulus (2010) argues that the intracranial electric field induced by typical tACS studies is below published thresholds of retinal sensitivity.

In a further attempt to identify the visual cortex as the site of interaction between tACS and the visual system, Kanai et al. (2010) delivered TMS impulses to the visual cortex while tACS was applied to the posterior part of the brain at different frequencies. The threshold needed to evoke a phosphene via TMS was recorded depending on tACS frequency. The results demonstrated that excitability is modulated by tACS in a frequency-dependent manner with maximal excitation at 20 Hz stimulation frequency as indexed by lowest phosphene thresholds. While this finding does not rule out a retinal origin of phosphenes for the previous study (Kanai et al., 2008), it supports the hypothesis that tACS modulates excitability of the visual cortex.

#### Visual, auditory, and somatosensory processing

In a visual study on contrast perception, Laczó et al. (2012) applied tACS in the gamma range (40, 60, 80 Hz) with the stimulation electrode (4 × 4 cm) over the central visual cortex and the reference (7 × 4 cm) over the vertex. Using a stimulation current of 1500 μA, the maximum current density in the stimulation electrode was 0.94 A/m^2^. Participants had to detect stationary random dot patterns in a four-alternative forced-choice paradigm. Results revealed that contrast sensitivity was not modulated by tACS, whereas contrast discrimination thresholds decreased during 60 Hz tACS relative to sham stimulation, but not during 40 or 80 Hz.

Brignani et al. (2013) presented leftward or rightward tilted low-contrast Gabor patches for 30 ms within the left or right visual hemifield, while participants received either sham stimulation or tACS at 6, 10, or 25 Hz with 1000 μA intensity (maximum current density in the electrode: 0.63 A/m^2^). The stimulation electrode (16 cm^2^) was placed over the left or right parietal-occipital regions and the reference (35 cm^2^) over the vertex. Participants had to report whether a Gabor patch was present or not (detection task) and whether it was tilted to the left or right (discrimination task). It was hypothesized that entraining alpha oscillations via 10 Hz tACS would increase the inhibitory alpha-effects at the target region of the stimulated hemisphere, thereby decreasing the accuracy in perceiving stimuli presented in the contralateral hemifield. Although the results demonstrated the expected accuracy decrease for 10 Hz tACS compared to sham and 25 Hz tACS, this effect was only found for the detection task and it was not hemifield-specific. The lack of hemispheric specificity might be due to the bi-hemispheric reference electrode. Moreover, the accuracy effects obtained with 10 Hz tACS did not differ significantly from those of 6 Hz tACS, leaving the issue of frequency specificity uncertain.

In an auditory detection paradigm, Neuling et al. (2012a) revealed dependencies between auditory detection performance and the phase of alpha oscillations over the temporal cortex. Participants were stimulated with otDCS at 10 Hz (DC of 1000 μA, modulated by a sinusoidal current of 425 μA) while having to detect a 500 Hz tone embedded in white noise at seven different signal-to-noise ratios (ranging from −4 to 8 dB). Electrodes were placed at temporal locations (cathode over left temporal cortex; anode over right temporal cortex). Results indicated that detection thresholds were modulated by the phase of the otDCS stimulation, demonstrating a causal link between oscillatory phase and perception. Furthermore, alpha power in the spontaneous EEG after stimulation was significantly increased relative to pre-stimulation alpha power, replicating the results of Zaehle et al. (2010).

Feurra et al. (2011b) studied the frequency-dependency of tactile sensations induced by tACS. The stimulation electrode (3 × 4 cm) was placed over the right somatosensory cortex, the reference electrode (5 × 7 cm) over the left posterior parietal cortex. The stimulation intensity of 1500 μA resulted in a maximum current density of 0.63 A/m^2^ in the stimulation electrode. Participants were stimulated at 35 different frequencies ranging from 2 to 70 Hz in randomized order for 5 s each and had to rate the presence and intensity of tactile sensations in their left hand. Results showed that stimulation in the alpha (10–14 Hz) and high gamma (52–70 Hz) range was significantly more effective in eliciting tactile sensations than stimulation in the delta (2–4 Hz) or the theta (6–8 Hz) range. Furthermore, beta stimulation (16–20 Hz) was more effective than that in the theta range.

Together, most of the studies on sensory processing demonstrate frequency-dependent perceptual consequences of tACS within different modalities and, thereby, the effectiveness of tACS in modulating ongoing rhythmic brain activity. However, the study by Brignani et al. (2013) represents a case of uncertain frequency specificity, i.e., yielding expected null results for one but not for another control frequency. Therefore, these findings are neither evidence against nor in favor of the possibility of tACS to modulate brain oscillations. In addition to frequency, the study by Neuling et al. (2012a) underlines the importance of oscillatory phase in entraining brain oscillations via tACS.

### Higher cognitive processes

#### Memory

Anodal otDCS has been used to study the functional roles of different brain oscillations in the formation of declarative memories during sleep and wakefulness. Marshall et al. (2006) focused on the association between slow oscillatory brain activity (<1 Hz) and sleep-dependent memory consolidation. After a learning period, participants were stimulated bilaterally at frontolateral locations with otDCS at 0.75 Hz (maximum current density in electrode: 5.17 A/m^2^) to boost slow oscillations that occur naturally during non-rapid eye movement (non-REM) sleep. Stimulation was applied for five 5-min periods separated by 1 min intervals without stimulation, during which EEG activity was analyzed. The results demonstrated a stimulation-induced increase of slow wave sleep (SWS) during the stimulation-free epochs, as reflected by an EEG power increase in the 0.5–1.0 Hz band. Slow frontal spindle activity (8–12 Hz) was also enhanced. On the behavioral level, the memory improvement after sleep compared with evening performance before sleep was stronger following otDCS than sham stimulation. Furthermore, both the electrophysiological and behavioral effects were frequency specific, since otDCS at 5 Hz (theta-tDCS) did not improve memory and reduced the power of slow oscillations.

Recently, the impact of theta-tDCS on memory consolidation and EEG activity has been investigated in more detail (Marshall et al., 2011). Using the same experimental setup as Marshall et al. (2006), theta-tDCS during non-REM sleep impaired memory consolidation and reduced both slow oscillations and frontal spindle activity. Thus, the theta-tDCS results were opposite to the effects induced by slow oscillatory stimulation, but they replicated the findings of the control condition using otDCS at 5 Hz from Marshall et al. (2006). Whereas these findings support a functional role for these oscillations in sleep-dependent memory consolidation during non-REM sleep, applying theta-tDCS during REM sleep did not affect consolidation, but produced a strong and widespread increase of gamma (25–45 Hz) power. These findings indicate a synchronizing effect of the theta rhythm on gamma oscillations that has no direct impact on memory consolidation during REM sleep (Marshall et al., 2011).

Whereas the study by Marshall et al. (2006) demonstrated a causal role of slow oscillations in declarative memory consolidation during sleep, Kirov et al. (2009) examined the impact of the same otDCS protocol on EEG and memory when applied during wakefulness. In analogy to the Marshall et al. (2006) study, stimulation of the participants started ~20 min after the end of the learning period and EEG was recorded until 1 h after stimulation has ended. Recall performance was tested after a 7 h retention period following learning. Electrophysiologically, otDCS at 0.75 Hz induced an EEG power increase in the slow oscillation frequency band that was restricted to frontal sites, however, the most pronounced and widespread power enhancement was found in the theta band (4–8 Hz). At the behavioral level, stimulation of the waking brain had no effect on memory consolidation after learning. Interestingly, when Kirov et al. (2009) applied stimulation during the learning period, i.e., while the material had to be encoded, learning performance improved as assessed by immediate recall performance.

Together, these studies demonstrate that the effects of otDCS on oscillatory EEG activity and related memory processes depend critically on the prevailing brain-state, i.e., whether stimulation was applied during wakefulness (Kirov et al., 2009), non-REM sleep (Marshall et al., 2006), or REM sleep (Marshall et al., 2011). A similar brain-state dependency of oscillatory brain stimulation has been shown for the visual (Kanai et al., 2008) and motor domain (Bergmann et al., 2009).

Using a working memory task, Polanía et al. (2012) tested the relevance of fronto-parietal theta phase-coupling for cognitive performance. In a first EEG experiment, the authors found an increase of phase synchronization between left frontal and parietal electrode sites at 4–7 Hz during memory matching. Furthermore, reaction times in the matching periods were faster when the phase lag between frontal and parietal oscillations was near to 0°. In a subsequent tACS experiment, Polanía et al. (2012) stimulated this fronto-parietal network with an oscillatory current at 6 Hz with a relative 0 or 180° phase difference or applied sham stimulation. As hypothesized by the authors, reaction times decreased during synchronization of fronto-parietal regions with 0° phase lag and increased during desynchronization with 180° compared to sham stimulation. Applying tACS at a control frequency of 35 Hz had no effect. These tACS results provide causal evidence for the relevance of theta phase-coupling during cognitive performance in a working memory task.

#### Ambiguous perception

**Figure 7:**
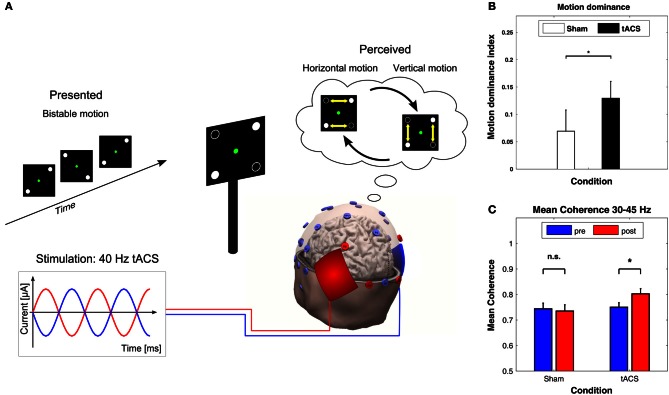
**Effects of 40 Hz tACS with 180° phase difference between hemispheres. (A)** Configuration of the bistable apparent motion display together with the EEG and tACS electrode montage. EEG electrodes that were used for analyzing interhemispheric coherence are indicated in red. The tACS sponge electrodes were placed bilaterally over the parietal-occipital cortex. This montage leads to 40 Hz stimulation with 180° phase difference between hemispheres. **(B)** The motion dominance index is significantly enhanced during 40 Hz tACS (black bar) as compared to sham stimulation (white bar), indicating that 40 Hz tACS results in a longer total duration of perceived vertical motion (^*^P < 0.05). Error bars display the standard error of the mean. **(C)** Mean coherence within the 30–45 Hz frequency band shows a significant increase from pre-tACS to post-tACS (right), but not from pre-sham to post-sham (left). Error bars correspond to standard errors of the mean; ^*^P < 0.05. Adapted from Strüber et al. (2013) with permission of the authors.

In a recent study with ambiguous visual stimuli, Strüber et al. (2013) applied 40 Hz tACS over occipital-parietal regions of both hemispheres while bistable apparent motion stimuli were presented which can be perceived as moving either horizontally or vertically (Figure 7A, top). In this paradigm, the switch between horizontal and vertical apparent motion is likely to involve a change in interhemispheric functional coupling. When 40 Hz tACS was applied with 180° phase difference between hemispheres (Figure 7A, bottom), the proportion of horizontal motion perception decreased significantly compared to sham stimulation (Figure 7B). Furthermore, EEG was recorded offline, i.e., 3 min before (pre-tACS) and after (post-tACS) applying tACS. After tACS, the interhemispheric gamma-band coherence increased between left and right parietal-occipital electrodes as compared to pre-tACS. This was not the case for sham stimulation (Figure 7C). Interestingly, when 40 Hz tACS was applied with 0° phase difference between hemispheres or with a control frequency of 6 Hz no behavioral or EEG-effects were observed (not shown here). These results were interpreted as evidence in favor of a causal role for gamma band oscillations in the perception of bistable apparent motion stimuli. It was further hypothesized that the external desynchronization of gamma oscillations via 40 Hz tACS with 180° interhemispheric phase difference might impair interhemispheric motion integration by a functional decoupling of the hemispheres.

This study by Strüber et al. (2013), together with the above-mentioned findings of Polanía et al. (2012), demonstrates that tACS can be used to couple or decouple inter-areal oscillatory activity either between or within hemispheres, which strongly supports the role of phase synchronization for large-scale neuronal integration (Engel et al., 2001; Varela et al., 2001; Siegel et al., 2012).

#### Decision-making

Sela et al. (2012) applied theta tACS to either the left or right dorsolateral prefrontal cortex (DLPFC) while participants performed a task that requires decision-making under risk. The rationale was to examine the laterality effects on risk-taking behavior. Stimulation was delivered during the task for 15 min (starting 5 min before task) using tACS with a frequency of 6.5 Hz and an intensity of 1 mA. One group of participants received tACS over the left hemisphere, one over the right, and another group received sham stimulation. EEG was not recorded. Only left hemispheric stimulation resulted in a significant effect on behavior, in that participants adopted a riskier decision-making strategy compared to right hemispheric stimulation and sham. According to the authors, these findings demonstrate a causal influence of both the DLPFC and theta oscillations on decision-making style. However, Sela et al. (2012) did not apply a control frequency, leaving the issue of frequency-specificity of the reported effects unaddressed. In this context, Feurra et al. (2012) pointed out that the inclusion of other frequencies that have been related to risky decision-making could have changed the pattern of results.

## Open questions/future perspectives

The reviewed studies are rather heterogeneous with respect to the experimental design and subsequent results. In the following sections, we critically discuss experimental parameters, as well as giving suggestions to overcome major concerns with regard to the validity of the results of tACS studies.

### Stimulation frequency

If the goal of a study is to demonstrate that tACS can modulate brain oscillations, the stimulation frequency should coincide with an existing brain oscillation, i.e., should be applied in a frequency range from delta (~0.5–4 Hz) to high gamma (~200 Hz). If a further goal of the study is to demonstrate that tACS can modulate a cognitive process which is associated with a certain brain oscillation, the stimulation frequency should match the brain oscillation that has been reported to correlate with a cognitive process. Since EEG frequencies vary inter-individually, this may require to adapt the stimulation frequency to the individual frequency that needs to be determined via EEG as in the case of stimulation at participants' IAF (Zaehle et al., 2010).

### Stimulation intensity

In the past, two procedures have been used to address the problem of stimulation intensity. Either all participants were stimulated at the same intensity or intensity was adapted to an individual threshold (e.g., phosphene or somatosensory threshold). Both procedures have certain advantages and disadvantages. If all participants are stimulated at the same intensity, this reduces the effort for determining individual thresholds, but, on the other hand, makes it possible that some participants sense the stimulation (via skin sensation or phosphenes), whereas others do not. This could, in principle, introduce a confound since more sensitive participants would be able to differentiate between stimulation and sham blocks, whereas less sensitive participants would not. To tackle this issue, two procedures have been established that conceal which block is currently performed. The first procedure is to fade-in the stimulation amplitude over a time interval of ~30 s. This reduces the skin sensations and has been applied frequently in tDCS studies where it is referred to as ramping-in. The second procedure consists of a short stimulation period at the beginning of a sham block which is faded out after ~30 s. This procedure mimics a stimulation block, because the stimulation is usually not felt consistently but only during the first seconds following the start of a stimulation block. When stimulation intensities are adapted to individual thresholds, confounds due to phosphenes or skin sensations can be excluded as alternative explanations for any observed effects. An obvious disadvantage of this method is that the intracranial current density can vary considerably across subjects. This problem, however, applies to both procedures as different skull thicknesses may also result in a significant variation of intracranial current densities. An ideal solution would be to acquire individual MR images in order to perform finite element modeling for each participant. Of course, this would require great effort both in terms of measurement time and computation time. Thus, if modeling is not feasible, the pros and cons of the different procedures have to be carefully balanced.

### Electrode montage

As indicated above, modeling studies have demonstrated that current flow is not always maximal underneath the stimulation electrode. In addition, new montages with multiple small electrodes offer the advantage of more focal stimulation as compared to two large electrodes. However, small electrodes also make skin sensations more likely due to increased current density if the intensity is kept constant. Again, the ideal solution would be to acquire individual MR images and to determine where to place electrodes based on the desired target region within the brain. At least two tools are currently freely available that allow this: SIMNIBs (http://simnibs.org) and Bonsai (http://neuralengr.com/bonsai). If this is not feasible, it would be desirable to compare the intended electrode montage with published modeling studies. For example, Neuling et al. (2012b) reported intracranial current density distributions of multiple electrode montages that have previously been used in cognitive experiments and therapeutic applications.

### Control conditions

A hitherto unsolved question is how to design an optimal control condition. Such a control or placebo condition should be identical to the stimulation or verum condition with respect to treatment duration, all possible sensations, time of day, experimenter, etc. but should not achieve the same cognitive or therapeutic effect. In addition, it would be desirable to carry out the two conditions in a double-blind procedure, i.e., neither experimenter nor participant know whether the verum or placebo stimulation is applied. One approach to achieve this goal would be to adapt stimulation intensities to be below certain thresholds for each participant—thus assuring that neither verum nor placebo stimulation can be sensed. However, as noted above, this results in significant variation of stimulation intensity across participants which is undesirable for comparable effects. Therefore, another approach is to apply identical stimulation intensity in all participants above threshold. In that case, participants will sense the onset of stimulation in both conditions. In the placebo condition, however, the stimulation will be ramped down after a few seconds. If the only goal of a study were to demonstrate that tACS has an effect as compared to no stimulation, the placebo condition could be sham stimulation. If, however, frequency specificity of tACS effects were to be demonstrated, the placebo conditions need to be a tACS stimulation at different frequencies. The study by Brignani et al. (2013) raises the question for appropriate control frequencies, since the use of multiple control frequencies was only partially successful. Ideally, the placebo conditions should apply two frequencies above and below the frequency of the verum condition demonstrating that the cognitive effect is absent or diminished at those control frequencies (Thut et al., 2011). Importantly, the frequency of the placebo condition should not be related to other cognitive effects such as memory which might be involved in the cognitive process at hand. It has to be noted, however, that currently no clear procedure has been established that defines the number of control frequencies or the distance in Hertz from the frequency of the verum condition in order to unequivocally demonstrate frequency specificity.

## Conclusion

An increasing number of studies on sensory, motor, and even higher cognitive processing demonstrates the effectiveness of tACS in modulating ongoing rhythmic activity in the human brain which, in turn, affects behavior. Interestingly, it has been demonstrated that, in addition to amplitude and frequency, also oscillatory phase plays a crucial role. Our understanding of the electrophysiological mechanisms of tACS has profited enormously from recent animal studies and computer simulations. In addition, realistically shaped models of the human head and brain have been successfully applied to further our knowledge of the intracranial current flow induced by tACS. Until recently, associations between cognitive processes and brain oscillations have been established via correlation. Using tACS offers the unique possibility to demonstrate a causal link between brain oscillations of a specific frequency and a specific cognitive process. If the brain oscillation is manipulated, the associated cognitive function is expected to co-vary. In case such co-variations can be demonstrated, a causal role of the oscillatory process must be assumed for the associated cognitive process. Recordings in animals have demonstrated convincingly that sinusoidal currents can entrain endogenous brain oscillations. However, so far, simultaneous recordings of EEG during tACS were not feasible due to strong artifacts. For future investigations, it would be worthwhile to combine electrophysiological recordings with tACS in order to shed further light on the neural mechanisms of brain entrainment.

### Conflict of interest statement

The authors declare that the research was conducted in the absence of any commercial or financial relationships that could be construed as a potential conflict of interest.

## Footnotes

^1^Note, that intracranial current density refers to current flow in cortex and is different from electrode current density as reported elsewhere in this article.

^2^Note, that here current density refers to the current density in the electrode and is computed by dividing the total stimulation current (e.g., 1 mA) by the area of the electrode (e.g., 35 cm^2^).
